# Editorial: Salt-sensitive hypertension: mechanisms, therapeutics, and beyond

**DOI:** 10.3389/fphar.2026.1790409

**Published:** 2026-02-18

**Authors:** Mohammad Saleem, Sepiso K. Masenga, Riyaz Mohamed, Jing O. Wu

**Affiliations:** 1 Department of Medicine, Division of Genetic Medicine and Clinical Pharmacology, Vanderbilt University Medical Center, Nashville, TN, United States; 2 Department of Cardiovascular Science and Metabolic Diseases, Division of Integrated Sciences, Livingstone Center for Prevention and Translational Science, Livingstone, Zambia; 3 Mulungushi University, Livingstone, Zambia; 4 Augusta University, Augusta, GA, United States; 5 Department of Physiology, College of Medicine - Tucson, The University of Arizona, Tucson, AZ, United States

**Keywords:** aldosteronism, HIV and sodium, inflammation, salt sensitive hypertension, salt-sensitivity and animal models, sodium and cardiac fibrosis, SSBP mechanism, vasculature

Excessive sodium intake continues to pose a significant global public health challenge and is a major contributor to hypertension and cardiovascular morbidity and mortality. Despite guidelines from leading organizations such as the American Heart Association (AHA) and the Centers for Disease Control and Prevention (CDC) to reduce sodium intake, consumption remains alarmingly high worldwide.

Almost half of the US adult population is hypertensive; about 50% of hypertensive and 25% of normotensive individuals exhibit salt sensitivity of blood pressure (SSBP). SSBP is defined as changes in blood pressure in response to sodium intake. SSBP is a risk factor for cardiovascular disease in both hypertensive and normotensive individuals (Sahinoz et al., 2021; Weinberger et al., 2001). Despite its clinical significance and several proposed mechanisms (Masenga et al., 2025; Saleem et al., 2024), the underlying pathophysiological mechanism of SSBP remains poorly understood. Current diagnostic approaches for SSBP are cumbersome and expensive, underscoring the need for simplified methods. Furthermore, there is no established biomarker for SSBP diagnosis, and no targeted therapeutic interventions are available to treat SSBP.

This research topic, “Salt-Sensitive Hypertension: Mechanisms, Therapeutics, and Beyond,” was conceived to address significant gaps in our understanding of SSBP, ranging from experimental findings to clinical insights. This editorial aims to showcase new mechanisms, advanced technologies, and potential treatments that connect the heart, kidneys, immune system, and brain. Collectively, the four articles in this Research Topic emphasize the complexity of SSBP and encourage a broader perspective beyond blood pressure alone.

First, Butler et al. present a broad, integrative review of the role of endothelial and vascular cell dysfunction in salt-sensitive hypertension (Butler et al.). This mini-review compiles studies and experimental animal models and examines how endothelial dysfunction, impaired autoregulation, inflammation, and immune cell trafficking contribute to SSBP and end-organ damage. Importantly, the review emphasizes sex-dependent mechanisms underlying salt sensitivity, an area of growing clinical and translational importance. By integrating endothelial biology with immune and vascular dysfunction, the article emphasizes that SSBP is a systemic disorder characterized by complex intercellular and organ-level interactions.

The original study by Mlejnek et al. provides critical mechanistic insights into salt sensitivity in primary aldosteronism over time (Mlejnek et al.). Using a well-established rat model, the authors identify early potassium depletion in skin and plasma as a precursor to significant sodium accumulation, hypertension, and the emergence of salt-sensitivity under high salt conditions. These findings challenge the conventional focus on sodium retention as the principal driver of aldosterone-mediated salt-sensitivity and highlight potassium imbalance as a key early contributor to the pathophysiology. Moreover, transcriptomic analyses of skin tissue reveal that electrolyte disturbances rapidly affect biological pathways involved in osmotic regulation and vascular function. This research highlights the importance of tissue electrolyte homeostasis in the development of salt-sensitive hypertension and opens new avenues for early therapeutic intervention targeting potassium regulation.

In addressing the deleterious consequences of SSBP, a comprehensive review by Mutengo et al. shift focus to the heart as a direct target of salt-induced injury (Mutengo et al.). Although salt sensitivity has traditionally been linked to blood pressure elevation, the review provides evidence for non-hemodynamic mechanisms by which high dietary sodium promotes myocardial fibrosis. The authors highlight redox-sensitive signaling, profibrotic pathways, and tissue sodium compartmentalization as key contributors to adverse cardiac remodeling. Importantly, the review discusses the emerging role of sodium magnetic resonance imaging (^23^Na-MRI) as a novel tool for visualizing myocardial sodium accumulation in humans. By linking salt sensitivity to diastolic dysfunction and heart failure with preserved ejection fraction, this work broadens the clinical relevance of SSBP and underscores the need for therapies that extend beyond conventional antihypertensive strategies.

Further expanding the scope of this Research Topic, the original research article by Masenga et al. presents important human data from a high-risk, understudied population (Masenga et al.). In a cohort of Zambian adults, people living with HIV (PLWH) consume significantly more dietary salt than healthy controls and show reduced salt taste perception. Notably, genetic variation in sodium- and sensory-related genes, including *TRPV1* and *SCNN1B*, was associated with salt intake, blood pressure, and body mass index. These findings suggest that gene-environment-disease interactions may influence dietary behaviors and cardiovascular risk in HIV populations. This study extends the concept of salt sensitivity beyond renal and vascular mechanisms to include sensory perception and genetic susceptibility, with important implications for personalized interventions in global health settings.

Together, these contributions highlight the multidisciplinary nature of recent SSBP research. They demonstrate that SSBP is not solely a reflection of sodium balance or blood pressure control but a complex phenotype regulated by endothelial function, systemic electrolyte homeostasis, tissue-specific sodium handling, and immune responses. These physiological processes are further shaped by genetic variants, sensory perception, and environmental factors (Liu et al., 2025). Importantly, the articles in this Research Topic also point to emerging diagnostic tools, such as sodium imaging, and therapeutic strategies that may include electrolyte modulation, anti-inflammatory approaches, and targeted pharmacologic interventions.

Despite these progress, substantial challenges continue to exist. A standardized clinical definition of salt sensitivity remains absent, diagnostic tools are limited, and targeted treatments are lacking. Future research should combine human phenotyping with mechanistic studies, utilize advanced imaging and omics technologies, and focus on population-specific vulnerabilities. Ongoing efforts in this field aim to discover biomarkers, improve risk assessment, and develop personalized strategies to reduce the cardiovascular and renal impacts of salt-sensitive hypertension.

In conclusion, this Research Topic advances our understanding of the mechanisms and consequences of SSBP and underscores the need for innovative, multidisciplinary approaches to address this persistent, clinically significant condition. We hope the insights presented here will stimulate further investigation and accelerate the development of effective diagnostic and therapeutic strategies for SSBP.
